# Prospective cohort study of dental implant success rate in patients with AIDS

**DOI:** 10.1186/s40729-016-0053-3

**Published:** 2016-09-28

**Authors:** Michael Clayton May, Paul Nielsen Andrews, Shadi Daher, Uday Nitin Reebye

**Affiliations:** 1 Triangle Implant Center, 5318 NC Highway 55, Suite 106, Durham, NC 27713 USA; 2 University of North Carolina, Chapel Hill, NC USA; 3 Boston University, Boston, MA USA

## Abstract

**Background:**

Oral health care of patients with acquired immune deficiency syndrome (AIDS) due to human immunodeficiency virus (HIV) is a growing area of concern, taking into consideration the increased life expectancy of patients resulting from antiretroviral therapy. There is insufficient literature regarding the impact of dental implants in AIDS patients. This study investigated the long-term clinical outcome of implant placement in patients diagnosed with AIDS.

**Methods:**

This monocentric study included AIDS patients with CD4 <200 cells/μL, age 18 years or older, and a minimum of one edentulous space requiring implant. All patients in the study were undergoing highly active antiretroviral therapy (HAART). HAART includes nucleoside reverse transcriptase inhibitors (NRTIs), non-nucleoside reverse transcriptase inhibitors (NNRTIs), protease inhibitors (PIs), and integrase strand transfer inhibitors (INSTIs). Typical treatment includes two different NTRIs, along with a third drug, either an INSTI, a PI, or an NNRTI. Bicon dental implants were placed in the patients after medical clearance and were followed up for 5 years. Bicon system implants were chosen because of availability and previous experience with this brand. Implant success criteria are defined as implants that had no clinical mobility at uncovering, no radiographic radiolucency, and allowed for loading and abutment placement. Implant success in AIDS patients was measured over a period of 5 years. Descriptive statistics were used.

**Results:**

Sixteen adults met the inclusion criteria (12 males and 4 females) with mean CD4 count as 141.25 (sd 35.5). Thirty-three implants were placed in selected patients. Average time to uncovering was 151 days (sd 25 days). Two of the three failures were maxillary implants in the anterior arch, and the third was in the mandibular posterior arch.

**Conclusions:**

The study found a slightly higher failure rate of 10 % in patients with AIDS, compared to widely accepted failure rates in healthy patients at 5–7 %. With the advent of new medical therapies, even AIDS patients should be offered the option of root-formed implants as a viable alternative to fixed and removable prosthetics.

## Background

The Joint United Nations Programme on HIV/AIDS estimates that 36.9 million (34.3–41.4 million) people are living with human immunodeficiency virus (HIV) infection [1]. In America, the Centers for Disease Control and Prevention (CDC) estimated that 1.2 million people aged 13 or older were HIV infected by the end of 2012 [2] and the cumulative population of persons surviving for more than 36 months after an acquired immune deficiency syndrome (AIDS) diagnosis to be 83 % [3]. As with the non-infected population, AIDS patients are in need of routine dental care, including implants. According to a 2015 systemic review, there have been only nine high-quality studies that have examined the implant outcomes in HIV-positive patients [4] and no studies looking at the implant outcomes in patients with the diagnosis of AIDS with a long-term follow-up. For this reason, a new prospective cohort study is needed. Although a great deal of research has been conducted in the pathophysiology, epidemiology, and treatment of AIDS, little is known with regard to the predictability of dental implants in this population. The purpose of this study is to evaluate implant outcomes in patients who have a diagnosis of AIDS, in order to provide some concrete data that may guide the dental practitioner and our medical counterparts when faced with treatment planning of these patients.

## Methods

Our study is a prospective study looking at the failure rates in root-formed implants in AIDS patients at 5 years post-surgical placement of the implant fixtures. Patients recruited for the study had to meet inclusion criteria which included diagnosis of AIDS measured by a pre-operative cluster of differentiation 4 (CD4) <200 cells/μL, age 18 years or older, and a minimum of one edentulous space requiring an implant as a viable restorative option. Exclusion criteria included current smokers, active periodontal disease, and non-restored remaining dentition. The study was conducted at a North Carolina community health center which serves a large group of patients infected with HIV, of which a substantial number met the CDC criteria for AIDS, CD4 <200 cells/μL [5]. All participants recruited for the study were patients of the health center under the care of the center’s HIV specialist and were patients of record of the center’s dental clinic. Patients did not receive any financial compensation for participating in the study. Internal review board approval was granted for this study.

Bicon® root-formed implants were placed in all patients. These implants were chosen because of availability and previous experience with this brand. All patients that met the inclusion criteria gave consent and had a pre-operative discussion on the risks associated with implant surgery. All cases were presented at implant rounds, and a comprehensive restorative work-up including panoramic and periapical radiographs, study models, and treatment plan was completed prior to surgical placement of any implants. Pre-operative medical work-up included medical clearance by the patients’ physician, CD4 counts, and viral loads. No perioperative antibiotics were given.

Post-operatively, all patients were placed on chlorhexidine gluconate 0.12 % rinse. No post-operative antibiotics were prescribed. All patients in the study were undergoing highly active antiretroviral therapy (HAART). HAART includes nucleoside reverse transcriptase inhibitors (NRTIs), non-nucleoside reverse transcriptase inhibitors (NNRTIs), protease inhibitors (PIs), and integrase strand transfer inhibitors (INSTIs). Typical treatment includes two different NTRIs, along with a third drug, either an INSTI, a PI, or an NNRTI. Patients on HAART and Pneumocystis pneumonia (PCP) prophylactic medications were told to continue their current regimen. All implants were placed by the same two dental surgeons, and all patients were followed by the center’s HIV/AIDS specialist. Patients were followed up at 1 week, 4 weeks, at uncovering (4–7 months), and then yearly till 5 years. The bone quality and consistency of this cohort did not differ from the same age and gender non-AIDS population. Infection, mobility, need for implant removal, non-bony union, and clinical mobility were considered to be implant failures. Implants with exposed threads were considered failures. Implant success criteria constituted implants that had no clinical mobility at uncovering, no radiographic radiolucency, and allowed for loading and abutment placement.

Standard descriptive statistics were used to examine the distribution of key variables (age, gender, CD4 count at baseline and follow-up, and failure rate) in the sample. Due to the small sample size in this study (*n* = 16), the relationship of CD4 count and failure will be examined graphically and cases which failed are discussed individually in the “Results” section. A bar chart is presented to examine the relationship between implant survival and CD4 count at baseline. A non-parametric survival curve (using the Kaplan-Meier method) is estimated for individual implants (*n* = 33), which depicts the relationship between implant survivorship and time since procedure.

## Results

**Fig. 1:**
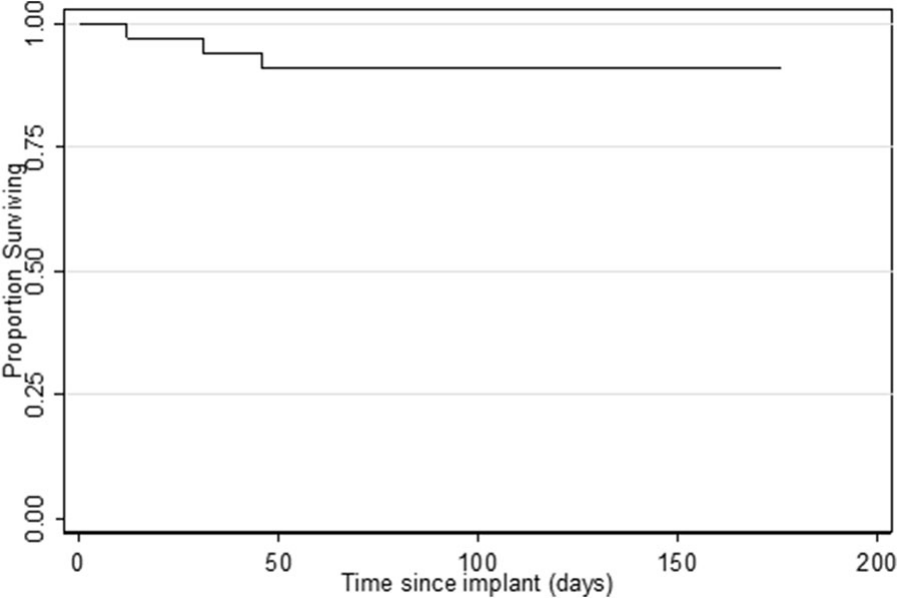
Kaplan-Meier survival curve for individual implants, *n* = 33

**Fig. 2:**
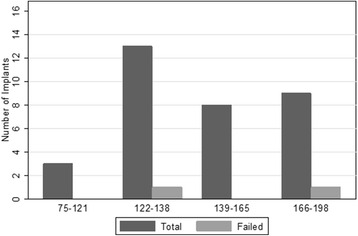
Number of implants and failures by quartiles of CD4

Descriptive statistics are presented in Table 1. Sixteen (*n* = 16) patients met our inclusion criteria and were included in our study. The sample included 12 males and 4 females. The mean age at enrollment was 36.2 years (sd 8.83 years). The mean CD4 count at the time of placement was 141.25 (sd 35.5). A total of 33 implants were placed in the 16 patients, including 5 mandibular implants and 28 maxillary implants. The average time to uncovering was 151 days (sd 25 days) for all implants that did not fail. Tables 2 and 3 present the distribution of implant site and implant size, respectively. A total of three implants, in two patients, failed prior to uncovering. This is shown graphically in Fig. 1. In the patient with one failure, the implant failed at post-op day 12 secondary to infection. In the patient with two failures, two failed secondary to non-bony union, at post-op days 31 and 46. Two of the three failures were maxillary implants in the anterior arch, and the third was in the mandibular posterior arch. A Kaplan-Meier survival function is shown in Fig. 2.

**Table 1:** Descriptive statistics (*n* = 16 patients)

Variable		Number	Mean (percentage)	Standard deviation
Age (years)		16	36.19	8.83
Sex	Male	12	(75)	–
	Female	4	(25)	–
CD4 count (cells/mm^3^)		16	141.25	35.5
No. of implants		33	–	–
No. of implants that failed		3	(9.1) (% failed)	–
Average time to failure (days)			29.7	–

**Table 2:** Frequency table of implant site

Implant site	Frequency	Percentage
15	2	6.06
14	1	3.03
13	3	9.09
12	4	12.12
11	7	21.21
21	4	12.12
22	1	3.03
23	3	9.09
24	1	3.03
25	2	6.06
35	1	3.03
33	1	3.03
43	1	3.03
45	1	3.03
46	1	3.03
Total	33	100

**Table 3:** Frequency table of implant size

Implant size	Frequency	Percent
4.5 × 11	7	21.21
4.5 × 8	1	3.03
4 × 11	10	30.3
4 × 8	13	39.39
5 × 11	1	3.03
5 × 8	1	3.03
Total	33	100

## Discussion

Since the AIDS epidemic reared its head in the 1980s, the nature of this disease has quickly evolved from a devastatingly debilitating disease to one of chronicity. These patients are requesting for and are entitled to the optimal restorative treatment plans, many of which include dental endosteal implants. Several authors have delved into the realm of implantology in the HIV-positive patient, but there is only one study specifically for the AIDS patient (CD4 count <200 cells/μL), though patients were followed up for 6 months only [6]. The two criteria generally used to ascertain the immunological status and disease progression of the HIV-positive patient are (1) viral load and (2) CD4 count. Viral load although controversial in its ability to quantify disease progression is stratified as high (5000–10,000 copies/mL), low (200–500 copies/mL), and as a treatment goal to be less than 50 copies/mL. The CD4 count has become the mainstay to our infectious disease colleagues to tailor the medicinal regiment of the HIV-positive and AIDS patient. The prophylactic medications administered are based upon the particular range of the CD4 count. This value is used as a window to predict the type of organisms the patient is susceptible to. We stratified our study population by these means in an effort to note any such trends.

As the HIV-positive patient reaches the low end of CD4 spectrum and manifests AIDS, this puts the patient in a further immunocompromised state, opening the doors to a multitude of opportunistic infections and neoplasia. One may erroneously hesitate to offer this patient the full scope of dental restorative options because of lack of awareness. Intuitively, one may expect this person to be more prone to infection, possessing a poorer quality of bone and compromised healing from surgery. These concerns may lead the dental surgeon to favor non-surgical restorations, prophylactic antibiotics, and a lower expectation of success if implants are to be placed.

Generally speaking, the use of antibiotics in dental implantology has been controversial. Amongst the reasons for early (preloading) implant failure are bacterial contamination, systemic disease, chemotherapy, overheating of bone, poor recipient site bone quality, and poor bone to implant contact upon surgery. After the prosthetic phase of the implant restoration, loading forces exceeding the bone to implant interface is an additional cause of early failure. Prophylactic antibiotics are shown to reduce dental implant failure but do not have much influence on post-operative infections [7]. The organisms most responsible for infections associated with the failing implant in a “healthy” patient are predominantly Gram-negative anaerobic rods [8].

Anecdotally, many practitioners have decided to administer prophylactic antibiotics to all of their patients receiving endosteal implants while others have taken a more conservative approach. A survey of 102 periodontists revealed that >50 % prescribe antibiotics in 10 specific periodontal or implant-related clinical circumstances [9]. A metaanalysis of patients HIV positive and with AIDS revealed no evidence of increased risk of complications associated with dental procedures [4].

HIV causes systemic infection with diverse multi-organ system manifestation, musculoskeletal symptoms often being the first clinical indication of the presence of disease. Habermann et al. in a study of 41 patients noted an increased infection rate of 12.7 % in HIV-positive hemophiliacs and non-hemophiliacs undergoing total joint arthroplasty. They also reported that there was no difference in functional outcomes in non-hemophilic HIV-positive and HIV-negative population after the surgery [10]. Supporting the above findings, a retrospective analysis of patients from 2003 to 2010 showed that none with CD4 indicative of AIDS at the time of total joint replacement developed implant infection [11].

In HIV-infected patients, CD4 count and albumin levels negatively correlate with incidence of post-operative sepsis, whereas surgical infections and previous major surgical procedures positively correlated with the incidence of post-operative sepsis [12]. Thirty-five HIV-infected patients undergoing abdominal operations with pre-operative CD4 <200 or CD4/CD8 ratio <0.15 had overall higher post-operative sepsis morbidity [13].

Regarding dental procedures, a retrospective cross-sectional study of 101 HIV patients was done from 2003 to 2005. Complication rate was found to be 2.2 % overall and 4.8 % after invasive dental procedures. No relationship was found between complications and immunological values [14]. Another study examining healing response after surgical crown lengthening in 21 patients with HIV was analyzed, and none had post-operative complications like delayed healing, infection, or prolonged bleeding [15].

In summary, our study indicated that dental endosteal implants placed in a population of AIDS patients under good surgical and prosthetic planning and surgical technique have no significant difference in failure rate than those placed in healthy patients. Therefore, this restoration should be made available to HIV-seropositive patients, including those patients meeting the criteria for an AIDS diagnosis. The success of the implants appears to be independent of CD4 count.

## Conclusions

Our study found a slightly higher failure rate of 10 % in patients with AIDS, compared to widely accepted failure rates in healthy patients at 5–7 %. A cohort sample size of 33 may be considered small for statistical power; however, the results from this study could lead to larger future prospective cohort studies with additional funding to recruit a larger cohort and comparison groups. The advent of new medical therapies has changed the face of HIV/AIDS in the western world. These patients live long and productive lives and should be offered the option of root-formed implants as a viable alternative to fixed and removable prosthetics.

## Abbreviations

AIDS: Acquired immune deficiency syndrome
CD4: Cluster of differentiation 4
CDC: Centers for Disease Control and Prevention
HAART: Highly active antiretroviral therapy
HIV: Human immunodeficiency virus
PCP: Pneumocystis pneumonia
